# A Review on the Multidisciplinary Approach for Cancer Management in the Eastern Mediterranean Region: A Focus on Nutritional, Lifestyle and Supportive Care

**DOI:** 10.3390/ijerph22040639

**Published:** 2025-04-18

**Authors:** Ayoub Al-Jawaldeh, Asmus Hammerich, Faisal Abdulghafar Aldayel, Giuseppe Troisi, Hanin Al-Jawaldeh, Hassan Aguenaou, Heba Alsawahli, Ibtihal Fadhil, Imen Sohaibani, Jalila El Ati, Jihan Azar, Lamia Mahmoud, Maha Barbar, Majid Mqbel Alkhalaf, Nahla Gafer, Taghreed Mohammed Alghaith, Zaynab Mahdi, Mandy Taktouk

**Affiliations:** 1 Regional Office for the Eastern Mediterranean, World Health Organization, Cairo 7608, Egypt; aljawaldeha@who.int (A.A.-J.); hammericha@who.int (A.H.); troisig@who.int (G.T.); alsawahlih@who.int (H.A.); azarj@who.int (J.A.); mahmoudl@who.int (L.M.); gafern@who.int (N.G.); mahdiz@who.int (Z.M.); 2 Saudi Public Health Authority, Riyadh 13352, Saudi Arabia; faadayel@pha.gov.sa (F.A.A.); issohaibani@pha.gov.sa (I.S.); mmkhalaf@pha.gov.sa (M.M.A.); tmghaith@pha.gov.sa (T.M.A.); 3 Action Against Hunger, Amman 11191, Jordan; haljawaldeh@yahoo.ca; 4 Department of Nutrition, International University of Rabat, Rabat 11103, Morocco; aguenaou.hassan@uit.ac.ma; 5 Regional Designated Center of Nutrition, AFRA/International Atomic Energy Agency, Kenitra 14000, Morocco; 6 NCD Alliance, Kuwait City 26733, Kuwait; ifadhil@hotmail.com; 7 SURVEN (Nutrition Surveillance and Epidemiology in Tunisia) Research Laboratory, INNTA (National Institute of Nutrition and Food Technology), Tunis 1007, Tunisia; jalila.elati@yahoo.fr; 8 Pediatrics Department, King Hussein Cancer Center, Amman 11941, Jordan; mbarbar@khcc.jo; 9 Nutrition and Food Sciences Department, Faculty of Agricultural and Food Sciences, American University of Beirut, Beirut 11-0236, Lebanon

**Keywords:** nutrition, lifestyle, supportive care, cancer care, management, multidisciplinary approach, Eastern Mediterranean Region

## Abstract

Cancer is one of the leading causes of global morbidity and mortality and one of the most challenging global health conditions, impacting the lives of millions every year. The Eastern Mediterranean Region (EMR) is not on track to achieve the sustainable development goal (SDG) target 3.4 which aims to reduce premature mortality (i.e., before the age of 70 years) for non-communicable diseases (NCDs), including cancer, by a third by year 2030; instead, it is projected that the EMR will experience the least progress towards achieving this target. This review therefore highlights the importance of context-specific cancer management, with a focus on nutritional, lifestyle and supportive care, in the EMR. A comprehensive literature search was conducted using electronic databases, including PubMed, Scopus and Google Scholar, as well as the Academy of Nutrition and Dietetics and key oncology institutes. Nutritional and lifestyle management is a fundamental aspect of cancer care which should be context-specific, achievable and individualized to minimize symptoms and side effects, while maximizing benefits and better addressing the needs of the patients with cancer. A multidisciplinary approach that integrates medical, nutritional, psychological and palliative care is essential to address this growing issue effectively. Cancer care and management requires coordinated efforts from policymakers, healthcare providers and communities to implement evidence-based interventions and promote cancer awareness.

## 1. Introduction

Cancer is one of the leading causes of global morbidity and mortality and one of the most challenging global health conditions, impacting the lives of millions every year [1,2]. It is a complex condition in which the irregular and uncontrolled growth and proliferation of certain cells interferes with normal tissue function and has the potential to spread throughout the body, which is known as metastasis [3,4,5]. The proposed etiology of cancer is multifactorial, whereby it stems from multiple interactions between genes, environmental exposures, lifestyle choices and sometimes infections [6,7]. Commonly known risk factors of cancer include genetics, physical carcinogens (radiation—ultraviolet and ionizing), chemical carcinogens (N-nitroso compounds (NOCs) (nitrosamines and nitrosamides) as a result of food processing, smoking and tobacco use, alcohol consumption and chemical exposures to specific substances such as bisphenol A, phthalate, heavy metals, insecticides, pesticides, herbicides, asbestos, aflatoxins, vinyl chloride, acrylamide, arsenic, benzene, cadmium, formaldehyde), biological carcinogens (infections, hormones and reduced immunity) and other factors (overweight and obesity, sedentary lifestyle and stress); with the impact of these factors increasing with age [2,8,9,10,11,12].

### 1.1. Types of Cancer

While cancer may develop in almost any part of the body, there are more than 200 types of cancer that are typically classified according to the tissue and site of origin [13]. Box 1 below summarizes the common categories [3,13,14].

Box 1 **Common categories of cancer.**

▪ Carcinomas: begin in epithelial cells that line internal and external lining of the body; examples include skin, breast, lung, colon, prostate and bladder cancer [15].
▪ Sarcomas: originate from connective or supportive tissues such as bones, tendons, cartilage, muscles, fat or blood vessels [16].
▪ Leukemia: cancer of white blood cells (WBCs); begins in tissues that make blood cells such as the bone marrow and lymphatic system [17].
▪ Lymphomas: cancer in the lymphatic system whereby they begin in lymph nodes and spread to other organs; examples include Hodgkin and non-Hodgkin lymphoma [18].
▪ Myelomas: cancer in the plasma cells of the bone marrow and the cells that make up the immune system [19].
▪ Melanomas: cancer in the skin and which arises from melanocytes, the cells that produce the melanin pigment in the skin.
▪ Central nervous system tumors: cancers originating in the brain and spinal cord [20].
▪ Mixed types: result from the combination of different cancer cell types, often involving both epithelial and mesenchymal cells, and may arise from a complex interplay of factors.

Globally, the most prevalent childhood cancers are leukemia (approximately 30%), brain tumors (26%), lymphomas (10–12%) and bone cancers (3–5%), due to genetic mutations, congenital predisposition syndromes and environmental influences that are common during childhood [21,22,23,24]. As for adults, cancers pertinent to the epithelial tissues and carcinomas are more prevalent; these include breast cancer among women, lung, colon, skin, digestive, endocrine, reproductive, respiratory and urinary types of cancer [25]. Within the Eastern Mediterranean region (EMR), and according to the Global Cancer Observatory database, the most common cancer types among women are breast, colorectal and liver cancer, while the most prevalent among men are lung, prostate, colorectal, liver and bladder cancer [26].

### 1.2. Global and Regional Prevalence

According to the International Agency for Research (IARC), by 2022, the global estimated age-standardized incidence rate was 196.9 (per 100,000), while that of mortality was reported at 91.7 (per 100,000) [26]. In the EMR, the reported age-standardized incidence rate was 127.2 (per 100,000), and that of mortality was 82.1 (per 100,000) in that year [26]. By 2050, the incidence and mortality rates are projected to increase by 126.4% and 143.4%, respectively, in the EMR [27]. However, these projections are lower than the majority of the other regions in the world whereby the increase in incidence ranged between 32.5 and 85.7% and that of mortality ranged between 43.9 and 96.9% in the Western Pacific, America, Europe and South-East Asia regions [27]. Africa, however, is expected to have a higher increase as compared to the EMR, whereby the projections show an increase of 147.2% and 153.3%, in incidence and mortality rates, respectively [27].

In 2015, the member states of the United Nations (UN) adopted the sustainable development goals (SDGs), including the SDG target 3.4 which aims to reduce premature mortality (i.e., before the age of 70 years) for non-communicable diseases (NCDs), including cancer, by a third by year 2030 [28]. However, the EMR is not on track to achieve the SDG target 3.4; instead, it is projected that the EMR will experience the least progress towards achieving this target [29,30].

**Figure 1:**
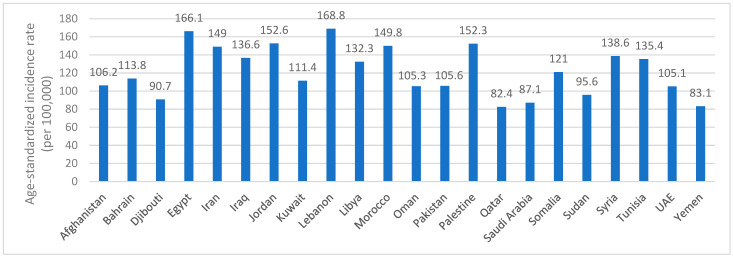
Estimated age-standardized incidence rate of cancer (per 100,000) by year 2022 in the EMR countries. Abbreviations: EMR: Eastern Mediterranean Region; UAE: United Arab Emirates.

Figure 1 shows the estimated age-standardized incidence rate of cancer in the EMR countries, by year 2022, and based on the IARC and World Health Organization (WHO) most recent data [26]. The estimated incidence rate ranged between 82.4 and 168.8 (per 100,000), whereby the lowest incidence rates were reported in Qatar (82.4), followed by Yemen (83.1), Saudi Arabia (87.1), Djibouti (90.7) and Sudan (95.6). The highest incidence rates were reported in Egypt (166.1) and Lebanon (168.8) [26] (Figure 1).

**Figure 2:**
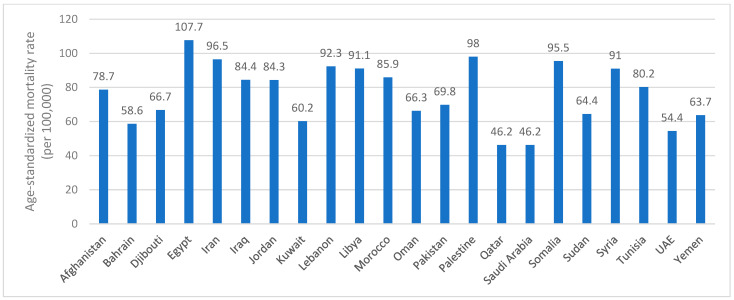
Estimated age-standardized mortality rate (per 100,000) by year 2022 in the EMR countries. Abbreviations: EMR: Eastern Mediterranean Region; UAE: United Arab Emirates.

Figure 2 shows the estimated age-standardized mortality rate in the EMR countries, by year 2022, and based on the IARC and WHO most recent data [26]. The estimated mortality rate ranged between 46.2 and 107.7 (per 100,000). The lowest rates of mortality were reported in Qatar (46.2) and Saudi Arabia (46.2), followed by the UAE (54.4), Bahrain (58.6) and Kuwait (60.2). The highest mortality rates were reported in Somalia (95.5), Iran (96.5), Palestine (98) and Egypt (107.7) [26] (Figure 2).

Table 1 shows the number of new cases and deaths (in thousands) in the EMR by 2022, and as projected for 2050, based on the type of cancer and as estimated by the IARC and WHO [26].

While lung cancer remains one of the most common cancers and the leading cause of cancer-related deaths worldwide, breast cancer contributed to the highest number of new cases and deaths by 2022 in the EMR, followed by lung cancer; pancreatic cancer and multiple myeloma contributed to the lowest numbers (Table 1) [26]. Table 1 also presents the projected number of new cases and deaths by 2050 in the EMR, which are subject to dynamic changes depending on regional variations (such as environmental exposures, healthcare infrastructure and socioeconomic conditions), lifestyle trends in the region (including obesity rates, sedentary lifestyle, physical activity levels, dietary habits and smoking habits) and medical advancements, all of which play a crucial role in shaping cancer incidence and mortality trends [26].

Nutritional, lifestyle and supportive care management play a critical role in comprehensive cancer care. However, in resource-limited settings such as the EMR, systemic and institutional challenges—including infrastructure constraints and healthcare access disparities—often hinder the integration of these essential interventions, thus highlighting the urgent need for long-term, sustainable solutions. Given that the EMR is not on track to achieve the SDG target 3.4 and that the region is projected to experience the least progress towards achieving this target, this review emphasizes the importance of nutritional, lifestyle and supportive care as part of cancer management in the EMR. Emphasis on management in the EMR is crucial to adapt it to the region’s needs due to ongoing political instability and conflicts which in turn impact the healthcare, resource availability, and access to advanced diagnostics and palliative care, faced in the region [31,32].

## 2. Materials and Methods

### 2.1. Search Strategy

A comprehensive literature search was conducted to identify relevant studies on cancer management. The search was performed using electronic databases, including PubMed, Scopus and Google Scholar, as well as the Academy of Nutrition and Dietetics and key oncology institutes. Keywords used included “cancer management”, “oncology treatment strategies”, “cancer medical management”, “radiation therapy”, “chemotherapy”, “immunotherapy”, “hormonal therapy”, “targeted therapy”, “hematopoietic cell transplantation”, “cancer treatment side effects”, “cancer nutritional management”, “cancer lifestyle management”, “nutritional support in cancer”, “enteral nutrition and cancer”, “parenteral nutrition and cancer”, “nutritional management and immunocompromised cancer patients”, “food safety and cancer”, “supportive care in cancer management”, “palliative care” and “multidisciplinary cancer approach”. The search was limited to peer-reviewed articles, reports, relevant nutritional recommendations, as well as guidelines published by the Academy of Nutrition and Dietetics and by key oncology institutes. Only English publications, since 2000, were included.

### 2.2. Data Synthesis

**Figure 3:**
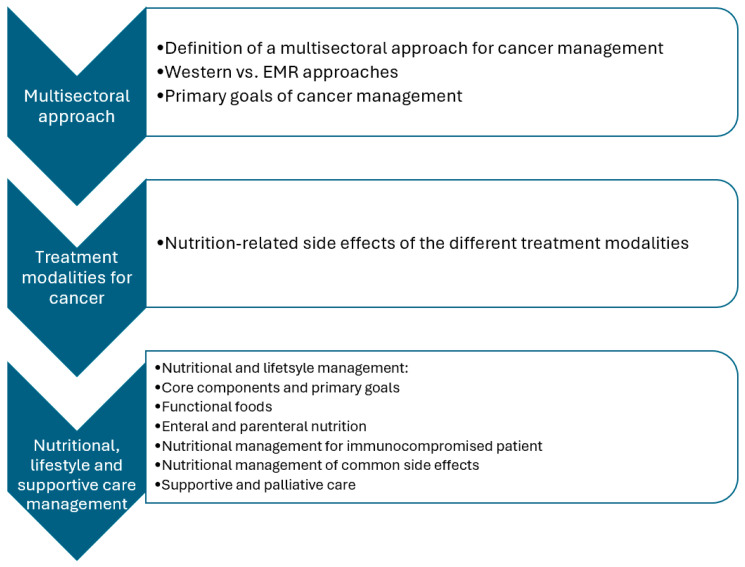
Summary of the main cancer management concepts in this review. Abbreviations: EMR: Eastern Mediterranean Region.

Relevant data were synthesized qualitatively and categorized into major concepts, as shown in Figure 3 below.

## 3. Management

Unlike the Western approach which is highly multidisciplinary, involving a collaborative effort between oncologists, pathologists, radiologists, nurses and palliative care specialists, many Eastern Mediterranean countries integrate traditional medicine (such as herbal remedies, acupuncture and spiritual practices) along with conventional Western medical treatments. While the Western world often employs the latest evidence-based protocols and technology, including precision medicine, immunotherapy and targeted therapies, several EMR countries face limitations in healthcare infrastructure and financial challenges, making access to modern technologies like precision medicine or newer immunotherapies less widespread [33,34,35]. The management of cancer depends on multiple factors including the type, stage and location of the cancer, as well as the age and overall health and nutritional status of the patient [36,37,38]. Effectively managing cancer involves a complex, multidisciplinary approach that combines nutritional and lifestyle adjustments, and supportive care.

The primary goals in cancer management are to remove or reduce the tumor, prevent its recurrence, manage symptoms, reduce unintentional weight loss and lean body mass loss, and improve the patient’s quality of life [25]. Moreover, the management should be individualized based on the state of the disease (i.e., whether in the malnourished or cachetic stage), whereby an optimal metabolic and immune balance is achieved for the patient under treatment [39]. Evidence has shown that proper therapy may reduce unplanned hospitalization, shorten hospital stays and enhance overall survival rates for patients undergoing cancer treatment [40,41,42,43,44,45,46].

Common adverse effects of the main treatment modalities for cancer are presented in Table 2 [33,47,48,49].

### 3.1. Nutritional and Lifestyle Management

Nutritional and lifestyle management is a fundamental aspect of cancer care that aims to sustain or improve food intake and alleviate metabolic disruptions, while preserving skeletal muscle mass and physical performance, and enhancing the overall quality of life [47]. Nutritional management plans should be context-specific, achievable and individualized to minimize symptoms and side effects, while maximizing benefits and better addressing the needs of the patients with cancer [25].

### 3.2. Core Components

The core components of nutritional management of cancer include the following: (1) providing education on malnutrition and dietary interventions to manage cancer and coexisting health conditions; (2) addressing malnutrition; (3) minimizing loss of appetite; (4) preventing or correcting nutritional deficiencies; (5) minimizing weight loss; (6) ensuring optimal energy, protein and fluids intake; (7) promoting proper food safety; (8) prescribing oral nutrition supplements (ONSs) if needed, (9) providing enteral or parenteral nutrition; (10) managing the nutritional side effects of the treatment; and (11) addressing cancer therapy interaction with food and dietary supplements [25,50,51].

### 3.3. Primary Goals

The primary goals of nutritional and lifestyle management for cancer patients include preventing and managing malnutrition, optimizing treatment tolerance and recovery, boosting immune function, and improving quality of life. Malnutrition is prevalent among cancer patients due to factors like reduced appetite, metabolic changes, medical therapy and use of medications, and the body’s increased need for nutrients to fight cancer [52]. Nutritional assessment and screening for malnutrition are recommended for all cancer patients at the time of diagnosis [49]. Preventing malnutrition by ensuring a balanced, varied and nutritious diet with adequate amounts of proteins, healthy fats, carbohydrates, vitamins, minerals and fluids is vital to preserve muscle mass, energy levels, overall body function and well-being, and thus reduce the likelihood of hospitalization [53,54,55]. Secondly, optimizing treatment tolerance and recovery, through personalized nutrition, symptom management and supportive care, is another key goal in cancer management. Adequate nutrition and sufficient provision of protein and energy are essential to reduce the potential side effects of treatments, help the body recover from the side effects between cancer treatment cycles and promote better health outcomes and prognosis. It is also crucial to incorporate physical activity and psychological support to enhance resilience, improve treatment outcomes and accelerate recovery [56]. Moreover, a balanced and nutritious diet, rich in vitamins, minerals and antioxidants, as well as regular physical activity and sleep, help support and boost immune health, reduce the risk of infections, reduce treatment complications and enhance recovery, specifically among immunocompromised cancer patients [57]. Finally, improving the quality of life among cancer patients involves comprehensive physical, emotional and social support. Proper nutritional support, pain management, mental health care and incorporating physical activity into the daily lifestyle, whenever possible, can provide better symptom management, maintain a sense of well-being and improve the patients’ strength and physical function, while also supporting their mental and emotional health, and improving their overall quality of life [58,59].

### 3.4. Functional Foods

Functional foods play a pivotal role in cancer management as they exhibit antioxidant, anti-inflammatory and immune-boosting properties. These compounds help reduce oxidative stress, neutralize free radicals and modulate signaling pathways involved in cancer progression [60]. Table 3 below lists the major food sources of the most common functional foods [25,60,61].

### 3.5. Enteral and Parenteral Nutrition

Criteria that determine the mode of nutrition support, whether enteral or parenteral, include the patient’s nutritional and health status, tumor location, GI function and overall prognosis. Among other contributing factors, patients consuming less than 60% of their energy needs for a period of 7–14 days, those in the intensive care unit (ICU) and not meeting their nutrient needs, and those with a specific clinical condition, limited nutrient absorption capacity or comorbidities, might need enteral or parenteral nutrition [62,63,64]. Enteral feeding is the primary intervention approach among the pediatric population to help promote proper growth and development, given that the GI tract is functioning properly [65]. Enteral feeding uses the digestive system which allows organs responsible for hormonal secretions to function normally, and thus avoids the flattening of the microvilli and prevents bacterial translocation [66]. Examples of enteral feeding tubes include nasogastric (from the nose to the stomach), nasoduodenal (from the nose to the duodenum), nasojejunal (from the nose to the jejunum), gastrostomy (directly to the stomach), jejunostomy (directly to the jejunum) and a combination of gastrostomy and jejunostomy [67]. Parenteral nutrition may be indicated only when enteral feeding is not well-tolerated for a period exceeding 7–14 days, and in the presence of abdominal surgery, chemotherapy, moderate-to-severe malnutrition, GI bleeding, inflammatory bowel diseases, intestinal obstructions, complete bowel failure and prolonged hospitalization. Otherwise, it is advisable to offer enteral nutrition, if feasible, to maintain the gut barrier, since it causes fewer infectious complications and is less costly, as compared to parenteral nutrition [47,49,62,68]. The burden and potential risks of both, enteral and parenteral nutrition, include being physically tethered to an apparatus, undergoing gastrostomy or central venous catheter placement, and the occurrence of complications related to the feeding apparatus [47]. In addition to this, parenteral nutrition may increase the risk of infections, injuries at the site of catheter insertion, blood clots, glucose imbalances, electrolyte imbalances, fluid overload, reduced GI function, liver and bone conditions [68]. Enteral and parenteral feeding should therefore be tailored to the patient’s nutritional needs, tolerances and lifestyle, as well as the ease of delivering the feeding in general [62].

### 3.6. Nutritional Management for Immunocompromised Patients

Patients who have undergone chemotherapy or hematopoietic cell transplantation might become immunocompromised and are therefore at an increased risk of infectious diseases that are (foodborne) transmitted through food and water. The table below summarizes the key components of nutritional support for immunocompromised patients (Table 4) [69,70,71,72,73,74,75,76,77].

Tips to minimize the risk of foodborne illnesses while shopping include purchasing cold and frozen items last to minimize their time outside the fridge or freezer, and then purchasing dry and shelf-stable products; taking a cooler with ice, insulated bags or any other cold source to safely transport the perishable foods back home, since temperatures above 4 °C can promote pathogen growth in perishable foods; checking vegetables and fruits for signs of bruising or molds; and avoiding products that have damaged packaging and canned goods that are dented [76]. Other tips to minimize the risk of foodborne illnesses when dining outside the home are asking the waiter, manager or chef if the meals or dishes contain any fish, poultry, meats or eggs that have not been appropriately cooked to a safe minimum internal temperature [76].

Moreover, immunocompromised patients should follow strict protective precautions at all times, by distancing themselves, wearing face masks and following proper hygiene practices.

### 3.7. Nutritional Management of Common Side Effects

Cancer treatments often lead to various side effects that can interfere with food intake, digestion and nutrient absorption. Based on the most commonly occurring symptoms and side effects among cancer patients, several nutritional management approaches and recommendations were shown to be effective according to scientific evidence [50,78,79,80,81,82,83,84]:

Reduced or altered dietary intake and poor appetite [85,86]:

- Consuming 5–6 smaller, nutrient-rich meals frequently, every 1–2 h, by adapting a schedule to promote adequate oral intake and diet tolerance.
- Establishing regular mealtimes by using a timer rather than relying merely on hunger cues.
- Avoiding meal skipping.
- Consuming the largest meal at the time of the day when appetite is at its peak.
- Keeping nutrient-dense foods such as yogurt, cheeses, nuts and smoothies, always available for snacking.
- Modifying food choices, preparation and presentation of foods and beverages, as needed.
- Trying new dishes and recipes from time to time.
- Having high-energy, high-protein meals and snacks, or considering high-energy and high-protein supplements and smoothies, if needed.
- Having juices or soups whenever solid foods are not well-tolerated.
- Drinking enough liquids. Consuming liquids between meals and throughout the day, rather than during mealtime.
- Developing a pleasant, relaxed and stress-free environment to promote a better meal experience with family, friends and colleagues.
- Engaging in light physical activity as tolerated.
- Offering appetite stimulants/medications when the intake of foods and beverages is not sufficient.
- Examining other factors that may have an impact on appetite, including stress, depression and medications.
- Considering enteral or parenteral nutrition, if needed.

Nausea and vomiting [87,88,89,90,91]:

- Consuming 5–6 smaller, frequent meals and snacks a day.
- Having clear liquids such as water, clear broth and electrolytes, for the first 24–48 h after treatment, and then shifting gradually to solid foods (examples include crackers, dry white toasts, cooked white rice or pasta, baked potato, grilled chicken, boiled and plain egg, plain yogurt).
- Avoiding meal skipping.
- Eating and drinking slowly.
- Ensuring the snacks and meals are cool or warm (yet properly cooked) as they may have less odor and are therefore better tolerated.
- Keeping foods covered to prevent exposing their odor.
- Avoiding foods with strong odors like fish, garlic, onions, or having an exhaust fan, or ensuring the patient waits in a distant, well-ventilated room while the food is being prepared.
- Considering dry, starchy foods (such as crackers, white toasts, dry cereals, mashed potatoes and white rice).
- Limiting the intake of acidic foods and beverages.
- Avoiding high-fat, oily, spicy or excessively sweet foods.
- Limiting fiber-rich foods (vegetables, fruits, whole grains, legumes, nuts and seeds) and gas-producing foods (such as cruciferous vegetables, raw vegetables including onions, cabbage, broccoli, as well as beans, dairy products with lactose and carbonated beverages).
- Drinking small amounts of clear liquids that are cold or at room temperature to make up for fluid losses.
- Drinking using a straw or directly from a closed cup or bottle to help avoid any odor and to increase the fluid intake.
- Sucking on sugar-free candies to help relieve nausea.
- Consuming bland, soft and easily digestible foods specifically on days of treatment.
- Incorporating complementary therapies such as ginger tea, deep breathing, acupressure bracelets and other relaxation approaches such as reading a book, listening to music and meditating, to aid in the management of symptoms.
- Rinsing the mouth with plain water or baking soda and salt throughout the day.
- Adhering to the prescribed medications for treating nausea.
- Using anti-emetics before meals to provide relief.
- Having the medications with the food rather than on an empty stomach.
- Considering IV fluids or parenteral nutrition when the patient presents with severe vomiting.

Diarrhea [92,93,94,95]:

- Consuming around 6–8 smaller meals and snacks every 3–4 h.
- Having the meals and snacks at room temperature as they may be more tolerated as such.
- Limiting the intake of high-insoluble fiber foods such as raw vegetables, bran, nuts and seeds.
- Eating high-soluble fiber foods such as pectin-rich fruits (apples, applesauce, bananas), oatmeal, soft white bread, potatoes and white rice; soluble fiber may be beneficial in managing diarrhea.
- Consuming bland foods such as plain rice cakes, plain noodles, plain white pasta, plain white toast, pretzels and crackers.
- Having hydrating beverages and foods like water, broth, gelatine and low-sugar popsicles.
- Avoiding lactose-rich foods and beverages such as dairy products and substituting them with lactose-free options such as unsweetened nut/seed milk and yogurt (soy, almond).
- Avoiding high-fat foods, fried foods, spicy foods and seasonings.
- Avoiding foods with sugar alcohols such as sugar-free candies and gums.
- Aiming for at least 8 cups of fluids a day.
- Limiting the consumption of juices because of their sorbitol content.
- Avoiding caffeine-rich beverages (such as coffee and tea), sugar-sweetened beverages, carbonated beverages and alcoholic drinks.
- Adhering to the prescribed medications for managing diarrhea.
- Having the medications with the food rather than on an empty stomach.
- Considering parenteral nutrition in case oral intake is not well-tolerated.

Sore mouth and throat [96]:

- Consuming foods that are easy to chew.
- Enjoying soft, moist foods with additional broth, soup, gravies, sauces, dressings or vegetable oils. Avoiding spicy, acidic, seasoned and salty options.
- Trying various food temperatures (warm, cool or chilled) to better identify which temperatures are most soothing and tolerable; yet ensuring foods are consumed at a safe temperature.
- Cutting foods into smaller pieces or blending them to ease swallowing.
- Preparing smoothies using low-acidic fruits such as melon, banana and peaches, and adding yogurt or milk if desired; they are good energy boosters.
- Sucking on low-sugar popsicles or ice chips to numb and ease the mouth or throat pain.
- Limiting the consumption of dry or rough-textured foods like dry toasts, crackers, pretzels, chips, nuts and raw vegetables and citrus fruits and juices.
- Avoiding vinegar, some condiments (pepper, chili, hot sauce), caffeine-rich beverages, carbonated beverages and alcoholic drinks.
- Avoiding foods that may cause irritations in the mouth and throat, such as crunchy foods, sugary foods, spicy foods, acidic foods and seasoned and salted foods.
- Increasing the intake of fluids.
- Drinking with a straw to minimize irritations in the mouth.
- Abstaining from smoking and using tobacco products.
- Rinsing the mouth with plain water or baking soda and salt, and avoiding alcohol-containing mouthwashes, throughout the day.

Fatigue [97,98]:

- Consuming smaller, frequent, easy-to-chew meals and snacks a day.
- Opting for ready-made foods, or foods that are easy to prepare and eat such as tuna, eggs, cheese and crackers, peanut butter and toast, cereal bars and puddings.
- Preparing additional foods, whenever it is most convenient.
- Keeping nutrient-rich foods always available for snacking.
- Having snacks and beverages by the bedside or chairside for easy access.
- Eating when appetite is at its peak.
- Drinking fluids, as much as possible.
- Limiting the consumption of caffeine-rich beverages and alcoholic drinks.
- Requesting support with meal preparation and grocery shopping, from family, friends and colleagues.
- Consulting with the healthcare professional regarding the need for supplements.
- Following good sleeping practices and having a short nap during the day if needed.
- Engaging in daily activities and exercises if tolerated.
- Consulting with a physical therapist for more guidance on exercises to help improve strength and mobility.
- Considering meditation, yoga and stretching exercises, whenever feasible.
- Consulting with the healthcare professional to help treat pain, anxiety or depression, if present.

Changes in taste and smell [99,100]:

- Consuming smaller, frequent meals and snacks a day.
- Educating patients on ways to change food texture, temperature or flavor in order to improve their intakes.
- Using plastic utensils when experiencing metallic tastes.
- Having cool foods rather than warm ones, as they may have less odor; yet ensuring foods are consumed at a safe temperature.
- Keeping foods covered to prevent exposing their odor.
- Avoiding foods with strong odors, or having an exhaust fan, or ensuring the patient waits in a distant, well-ventilated room while the food is being prepared.
- Choosing non-meat protein foods such as chicken, turkey, dairy products and legumes, particularly if meat aversions are being experienced.
- Enhancing the flavor of foods or even masking changes in taste by using sauces, marinades, herbs, spices, lemon juice and seasoning blends; yet avoiding the use of onions and garlic because of their strong odor.
- Using flavorings and seasonings to prepare foods.
- Enhancing the flavor of water by using herbs, lemons or fruits.
- Trying citrus fruits to promote saliva production, only in the absence of open sores.
- Enjoying tart foods such as lemon sorbet, citrus fruits, lemonade, dried cranberries, pickles and olives, which may be well-tolerated if sores are not present.
- Using salt to reduce the sweetness of sugary foods.
- Drinking using a straw or directly from a closed cup or bottle to help avoid any odor and to increase the fluid intake.
- Considering the foods and beverages that are more appealing and preferred by the patient.
- Practicing proper oral hygiene by brushing the teeth after meals with a soft toothbrush and by rinsing the mouth with plain water or baking soda and salt, and avoiding alcohol-containing mouthwashes, throughout the day.

Dry mouth/Xerostomia [101]:

- Enjoying soft, moist foods with additional broth, soup, gravies, sauces, dressings or vegetable oils. Avoiding hot, spicy, sour or salty options.
- Soaking foods in liquids to soften them.
- Choosing foods that can be easily swallowed.
- Sucking on low-sugar ice pops, frozen grapes or melon balls.
- Enjoying tart foods such as lemon sorbet, citrus fruits, lemonade, dried cranberries, pickles and olives, for better saliva production, if sores are not present.
- Consuming mashed rice and potatoes, instead of dry bread, toast or crackers.
- Considering liquid nutritional supplements to help meet nutritional needs.
- Limiting foods that may impact the mouth such as hard, hot, spicy, sour, salty or crunchy foods.
- Sipping small amounts of fluids between meals.
- Having a water bottle on-the-go, at all times.
- Avoiding caffeine-rich beverages and alcoholic drinks.
- Abstaining from smoking and using tobacco products.
- Avoiding cariogenic foods such as sweets, candies, cakes, sweet pastries, as well as sugar-sweetened beverages.
- Brushing the teeth after meals with a soft toothbrush and rinsing the mouth with plain water or baking soda and salt, and avoiding alcohol-containing mouthwashes, throughout the day.
- Applying lip balms to moisturize the lips.
- Using a cool mist humidifier when sleeping to increase the moisture in the air.
- Consulting with the healthcare professional regarding the need for medications that may help.

Chewing and swallowing difficulties, inadequate dental health and esophagitis [102]:

- Consuming 5–6 smaller, frequent meals and snacks a day.
- Modifying food texture, as tolerated.
- Thickening liquids if necessary.
- Cutting foods into smaller pieces or blending them to ease swallowing.
- Considering soft, moist or pureed foods if necessary.
- Adding broth, soup, gravies, sauces, dressings or vegetable oils to moisten dry foods and meals such as meats and cereals. Avoiding spicy and high-acidic options.
- Having high-energy, high-protein meals and snacks, or considering high-energy and high-protein supplements and smoothies, if needed.
- Limiting dry, sharp, crunchy or rough-textured foods such as dry toasts, crackers, pretzels, chips, nuts, raw vegetables and citrus fruits.
- Soaking some dry foods, such as breads or cereals, in milk may help soften them.
- Limiting the consumption of spicy foods, high-acidic foods and beverages such as tomatoes, oranges, lemonades, citrus fruit juices and carbonated beverages.
- Drinking cold beverages or beverages at room temperature.
- Drinking with a straw to minimize irritations in the mouth.
- Avoiding alcoholic drinks.
- Abstaining from smoking and using tobacco products.
- Practicing proper oral hygiene but avoiding alcohol-containing mouthwashes.
- Adhering to the prescribed medications for treating painful swallowing, oral discomfort, esophagitis and/or infection.
- Considering enteral nutrition if needed to provide sufficient intake as long as the patient is unable to consume adequate amounts orally.
- It is recommended to give bisphosphonates or send the patient to the dentist for full dental inspection and care before radiotherapy to the jaw.

### 3.8. Supportive Care in Cancer Management

Supportive care is a key component of comprehensive cancer management, whereby its multidisciplinary approach ensures that patients receive holistic support to maintain a good quality of life and end-of-life care [103,104].

Palliative care helps improve the quality of life of patients and their families by preventing and relieving their suffering through early identification, assessment and management of pain and associated complications, whether physical (symptoms as a direct effect of the disease or its treatment), psychological (altered body image due to disfigurement, stress, anxiety, depression and fear), social (loss of role in the community, feelings of isolation and financial challenges) or spiritual (such as guilt feelings or misinterpretations of suffering) [105,106,107]. Palliative care therefore aims to provide comfort to the patients within their environment, manage symptoms while integrating the physical, psychological, social and spiritual dimensions of patient care, minimize feelings of loneliness, stress, anxiety and fear, encourage patients to be independent whenever possible, while considering the patients’ social and cultural backgrounds, preferences and emotional conditions [105,108,109]. It also builds a supportive community to help families manage the patient’s situation and their own emotional status. Palliative care may also rely on essential medications and focus on non-pharmacological measures to support the maintenance of strength, vitality, overall well-being, independence and the ability to engage in daily physical activities [105,108]. It is therefore crucial that healthcare facilities are well-equipped with the required medications to ensure the contingent needs of the patients are met. The palliative care team usually includes palliative care specialists, primary physicians, caregivers, nurses, psychologists, pharmacists, social and community workers, spiritual care providers, counselors and nutritionists, in addition to the patient, family, friends and colleagues [110].

The palliative care team is essential in managing misunderstandings related to food or nutrition, (e.g., a cancer patient abstaining from certain food, or a patient with a colostomy bag reducing their food intake so as to reduce their waste). During the last days of life, discussions with the family need to take place to have them understand that the body is not going to benefit from forced feeding. The primary goal is to enhance quality of life while respecting the patient’s preferences in terms of foods and beverages. Common challenges such as decreased appetite, difficulty swallowing and nausea require personalized management strategies. Eating is not only a function but has good, strong cultural connotations in the EMR, especially when eating together as a family. Encouraging this habit brings the patient back to normality and reduces feelings of isolation. Ethical considerations involve engaging patients and families in discussions related to the goals of care, while respecting cultural and personal beliefs that influence nutritional choices, and carefully evaluating the benefits and burdens of artificial nutrition and hydration (ANH) [108,111].

### 3.9. Strengths and Limitations

This review is characterized by a number of strengths and limitations. It emphasizes cancer management with an adaptation to the EMR’s needs, a region that has faced ongoing political instability and conflicts, for decades. However, although every effort was made to integrate the nutritional, lifestyle and supportive care aspects of the multidisciplinary approach in this review, one of its limitations is the generalization of the challenges present in the region. In fact, the EMR encompasses various countries that differ in their healthcare infrastructures, resource availability, and access to advanced diagnostics, treatments and palliative care. Furthermore, this review was not exhaustive in its language coverage and hence articles, reports, relevant nutritional recommendations and guidelines that may be available in languages other than English would have been missed.

## 4. Conclusions

Cancer is a significant global health challenge, and its burden is rapidly increasing in the EMR. The region’s diverse socioeconomic and cultural contexts, coupled with disparities in healthcare access, present unique challenges in cancer management. A multidisciplinary approach that integrates medical, nutritional, psychological and palliative care is essential to address this growing issue effectively.

Cancer prevalence in the EMR is projected to double by 2050 [26], demanding immediate action to improve prevention, early diagnosis and equitable access to advanced treatments such as targeted therapies, immunotherapies and precision oncology. Addressing malnutrition and optimizing nutritional care, including lifestyle management, are critical in managing treatment side effects and improving patient outcomes. Personalized nutritional plans and strict food safety and hygiene measures are particularly important for immunocompromised patients to minimize infection risks during periods of vulnerability. Supportive and palliative care play a vital role in providing holistic support, addressing physical, psychological and social needs, and fostering dignity and comfort. Cultural sensitivity, including promoting family-centered practices, enhances care quality.

Cancer care and management require coordinated efforts from policymakers, healthcare providers (including oncologists, nutritionists, caregivers, nurses, psychologists and pharmacists) and communities to implement evidence-based interventions and promote cancer awareness.

## Figures and Tables

**Table 1:** Number of new cases and deaths in the EMR by 2022, and as projected for 2050, based on the type of cancer.

	Number of New Cases—2022 (In Thousands)	Number of Deaths—2022 (In Thousands)	Projected Number of New Cases—2050 (In Thousands)	Projected Number of Deaths—2050 (In Thousands)
Breast	130.1	52.8	401.4	186.4
Lung	54.7	49.4	133.8	125.4
Colorectal	54	30	128	79.3
Liver	48.6	46.8	117.9	116.5
Lymphoma	44.3	22.3	90.8	52.1
Gastric	37.8	31.3	101.9	87
Leukemia	34.5	25.3	65.9	54.7
Esophageal	19	17.9	46.7	46
Pancreatic	14.1	13.4	37	36
Multiple myeloma	7.3	6.1	18.3	16.4

Abbreviations: EMR: Eastern Mediterranean Region.

**Table 2:** Common adverse effects of the main treatment modalities for cancer.

Treatment Modality	Adverse Effects
Surgery	Common side effects of gastrointestinal (GI) surgeries include early satiety, diarrhea, constipation, nausea and vomiting.Common side effects of head and neck surgeries (particularly in the mouth and pharyngeal regions) include chewing difficulties and dysphagia.Other side effects include fatigue and altered bowel function.GI surgeries mandate nill oral intake post-surgery that could last a few days. Moreover, adverse effects are usually dependent on the area/organs being subject to surgery.
Radiation therapy	Radiation usually causes hair loss and skin changes at the irradiated place. If the radiotherapy is close to the abdominal area, diarrhea, vomiting and loss of appetite might happen. Radiation to the head and neck area leads to mucositis, dysphagia, xerostomia and alterations in taste. Changing the radiation dose or direction might help reduce these side effects.
Chemotherapy	Common side effects include early satiety, diarrhea, constipation, nausea, vomiting, fatigue, oral sores, alterations in taste, dysphagia, mucositis, infections, hair loss, anemia, loss of appetite, weight loss and anorexia.
Immunotherapy	Common side effects include diarrhea, constipation, nausea, vomiting, GI perforation, hemorrhage, loss of appetite and anorexia.
Hormonal therapy	Common side effects include diarrhea, nausea, vomiting, fatigue, edema and fluid retention, high cholesterol levels and hyperglycemia, increased appetite and weight gain.
Targeted therapy	Common side effects include diarrhea, oral sores and wound-healing difficulties.
Hematopoietic cell transplantation	Common side effects include diarrhea, nausea, vomiting, fatigue, xerostomia, oral sores, altered sense of taste, oral and esophageal mucositis, weight loss and anorexia.

**Table 3:** Major food sources of the most common functional foods.

Functional Food	Examples of Food Sources
Flavonoids	Apples, strawberries, blueberries, cranberries, dark green leafy vegetables, onions, garlic, whole grains, legumes, walnuts, coffee, tea, wine
Phenolic acids	Fruits, vegetables, whole grains, soybeans, walnuts, coffee, tea
Lycopene	Papaya, pink grapefruit, watermelon, apricots, tomatoes and tomato products
Alpha- and beta-carotenes	Mangoes, carrots, pumpkin, green leafy vegetables, sweet potato
Anthocyanins	Berries, grapes, plums, purple cabbage
Probiotics	Yogurt, fermented foods
Resveratrol	Berries, grapes, peanuts
Lignans	Whole grains, legumes, flaxseeds, coffee
Curcumin	Turmeric

**Table 4:** Nutritional support for immunocompromised patients.

General Recommendations	
▪ Avoiding raw fish, poultry and meats, raw or undercooked eggs (below a safe temperature), aged cheeses, soft cheeses and cheeses containing molds, unclean vegetables and fruits, and unpasteurized beverages (such as untreated water, unpasteurized milk beverages, vegetable juices and fruit juices). ▪ Ensuring that foods are properly cooked to a safe temperature, cooled and reheated to suitable temperatures. ▪ Preventing cross-contamination between raw foods (such as meats) and prepared or ready-made foods, in the refrigerator and when grocery shopping. ▪ Washing fresh vegetables and fruits thoroughly before consumption. ▪ Ensuring that the drinking water is from a reliable source. ▪ Avoiding the use of bulk bins, salad bars and buffets. ▪ Checking regularly the expiration dates on food products. ▪ Following the principle of “when in doubt, throw it out”. ▪ Washing the hands frequently. ▪ Keeping the kitchen surfaces and utensils clean at all times. ▪ Using alcohol-based sanitizers; this applies to the patients, their families and caregivers.	
Safe food practices	
Clean	▪ Washing raw vegetables and fruits thoroughly with clean running water and using a clean brush to scrub firm-skinned vegetables and fruits, including those with inedible skins and rinds. ▪ Avoiding green leafy vegetables and thin-skinned fruits such as berries, since they are difficult to clean enough. ▪ Cleaning the lids of canned products before opening them. ▪ Cleaning the hands regularly with soap and warm water for around 20 s prior to handling and eating food. ▪ Cleaning the cutting boards, utensils and kitchen surfaces with soap and warm water consistently between the preparation of different meals. ▪ Using paper towels, whenever possible. However, if using clothes or sponges, make sure to regularly wash them.
2. Separate	▪ Preventing cross-contamination by separating raw fish, poultry, meats and eggs from prepared or ready-made foods in the refrigerator and while grocery shopping. ▪ Placing raw fish, poultry and meat on the lowest shelf in the refrigerator so that their juices do not drip on other foods that have already been cooked. ▪ Avoiding the reuse of marinades unless they are properly boiled first. ▪ Using a separate cutting board for raw fish, poultry and meats and then using clean plates and utensils when offering them as cooked food. ▪ Using different cutting boards for vegetables and fruits. ▪ Making sure to properly clean the cutting boards following every use.
3. Cook	▪ Cooking food thoroughly until the safe internal temperatures are reached: 63 °C for fish, beef, veal and lamb; 72 °C for ground meats, casseroles and egg dishes; 74 °C for poultry and leftovers. ▪ Using a food thermometer to check the temperature when the food is cooked. Check the internal temperature on several locations on the food. ▪ Avoiding the consumption of meals and dishes that include raw fish or meats. ▪ Heating soups, gravies and sauces to a boil, which is approximately 74 °C. ▪ Covering, stirring and rotating foods when cooking in a microwave oven in order to ensure the food is cooked evenly. If necessary, the dish should be rotated by hand once or twice while cooking. Make sure the safe internal temperature is reached.
4. Chill	▪ Chilling perishable and cooked foods at a temperature below 4 °C; avoiding their storage at room temperature for a period of time that exceeds 2 h unless they are kept hot and at a suitable and safe temperature. ▪ Avoiding the thawing of foods at room temperature, such as on the kitchen countertop. Instead, thawing it in the refrigerator, under cold water or in a microwave is advisable. Once thawed, it is recommended to immediately cook the food. ▪ Using defrosted food right away and without refreezing them. ▪ Eating leftovers within 2 days. ▪ Consuming foods prior to their expiration date, as indicated on the products. ▪ Ensuring the temperature of the refrigerator and freezer are within the normal range at all times.

## Data Availability

The data presented in this study are available in the manuscript.

## Abbreviations

The following abbreviations are used in this manuscript:

ANH	Artificial nutrition and hydration
EMR	Eastern Mediterranean Region
GI	Gastrointestinal
IARC	International Agency for Research
ICU	Intensive care unit
NCDs	Non-communicable diseases
NOCs	N-nitroso compounds
ONSs	Oral nutrition supplements
SDGs	Sustainable Development Goals
UAE	United Arab Emirates
UN	United Nations
WBCs	White blood cells
WHO	World Health Organization
